# *CLCA2* expression is associated with survival among African American women with triple negative breast cancer

**DOI:** 10.1371/journal.pone.0231712

**Published:** 2020-04-16

**Authors:** Kristen S. Purrington, Jimmie Knight, Gregory Dyson, Rouba Ali-Fehmi, Ann G. Schwartz, Julie L. Boerner, Sudeshna Bandyopadhyay

**Affiliations:** 1 Department of Oncology, Wayne State University School of Medicine, Detroit, Michigan, United States of America; 2 Population Studies and Disparities Research Program, Barbara Ann Karmanos Cancer Institute, Detroit, Michigan, United States of America; 3 Department of Pathology, Wayne State University School of Medicine, Detroit, Michigan, United States of America; 4 Tumor Biology and Microenvironment Program, Barbara Ann Karmanos Cancer Institute, Detroit, Michigan, United States of America; 5 Molecular Therapeutics Program, Barbara Ann Karmanos Cancer Institute, Detroit, Michigan, United States of America; Florida State University, UNITED STATES

## Abstract

**Purpose:**

Black/African American (AA) women are twice as likely to be diagnosed with triple negative breast cancer (TNBC) compared to whites, an aggressive breast cancer subtype associated with poor prognosis. There are no routinely used targeted clinical therapies for TNBC; thus there is a clear need to identify prognostic markers and potential therapeutic targets.

**Methods:**

We evaluated expression of 27,016 genes in 155 treatment-naïve TN tumors from AA women in Detroit. Associations with survival were evaluated using Cox proportional hazards models adjusting for stage and age at diagnosis, and p-values were corrected using a false discovery rate. Our validation sample consisted of 494 TN tumors using four publically available data sets. Meta-analyses were performed using summary statistics from the four validation results.

**Results:**

In the Detroit AA cohort, *CLCA2* [Hazard ratio (HR) = 1.56, 95% confidence interval (CI) 1.31–1.86, nominal p = 5.1x10-7, FDR p = 0.014], *SPIC* [HR = 1.47, 95%CI 1.26–1.73, nominal p = 1.8x10-6, FDR p = 0.022], and *MIR4311* [HR = 1.57, 95% CI 1.31–1.92, nominal p = 2.5x10-5, FDR p = 0.022] expression were associated with overall survival. Further adjustment for treatment and breast cancer specific survival analysis did not substantially alter effect estimates. *CLCA2* was also associated with increased risk of death in the validation cohorts [HR = 1.14, 95% CI 1.05–1.24, p = 0.038, p-heterogeneity = 0.88].

**Conclusions:**

We identified *CLCA2* as a potential prognostic marker for TNBC in AA women.

## Introduction

Triple negative breast cancers are a distinct histopathologic subtype of breast cancer (BC) that accounts for approximately 15% of all invasive BCs [1, 2] where <1% of tumors cells stain positive for estrogen receptor (ER), progesterone receptor (PR), and human epidermal growth factor receptor-2 (HER2) protein expression. Clinical outcomes among TNBC patients have a unique pattern, including a peak risk of death and recurrence within the first three years following treatment [3] followed by a 50% decrease in risk beyond five years compared to hormone receptor-positive BC patients [4]. There are no routinely used targeted clinical therapies for TNBC or effective approaches for reducing high cancer mortality among this subtype, partly because we have yet to identify the underlying etiologic factors. Thus, there is a clear need to better identify the molecular processes in TN tumors related to clinical outcomes that could be explored as therapeutic targets.

Black/African American (AA) women are twice as likely to be diagnosed with TNBC compared to white/European American women [5]. Poor TNBC survival contributes to the racial disparity in overall breast cancer outcomes because are approximately 40% more likely to die from overall BC as white women, due in large part to the increased incidence of aggressive TNBC among AA women [6]. Several studies further suggest that AA women with TNBC specifically experience poorer clinical outcomes compared to white women [7–12], although this relationship is less clear. While AA women would receive substantial benefit from targeted TNBC therapies, few studies exist evaluating molecular processes related to survival specifically in TN tumors from AA women.

Evaluation of the gene expression profiles of TN tumors is a valuable tool for evaluating prognostic biomarkers. This was first robustly demonstrated through the identification of four main intrinsic breast cancer subtypes with important prognostic implications, now evaluated using the PAM50 expression microarray: two luminal epithelial groups (A and B); a HER2 over-expressing group; and a basal-like group that is largely TNBC [12, 13]. Although the majority of TN tumors are classified as basal-like (80–85%), they have been shown to have significant biological heterogeneity [14]. Consensus across multiple TNBC subtyping studies appears to classify TN tumors into 3 to 4 molecular subtypes: basal-like, immune enriched, mesenchymal (M), and luminal androgen receptor (LAR) [13]. With the exception of the immune-enriched subtype, which consistently is associated with better survival across studies, there is discrepancy in the prognostic implications across subtypes [14]. While expression of individual genes or gene signatures have been evaluated with respect to clinical outcomes [15–24], none of these are used clinically to identify patients at higher risk or recurrence or death or to guide treatment decision making. Thus, there remains a need to identify strong prognostic biomarkers in TNBC that can be used, particularly in AA women who have a higher burden of this aggressive breast cancer subtype. Here we evaluated associations between gene expression and survival in AA women with TNBC to identify potential prognostic factors and potential therapeutic targets.

## Methods

### Detroit AA cohort and sample selection

Inclusion criteria for the TNBC cohort required that participants were (1) African American, (2) female, (3) diagnosed with primary invasive breast cancer, (4) negative for ER, PR, and HER2, and (5) underwent surgery format the Karmanos Cancer Center in Detroit, MI from 2004–2013. Women meeting inclusion criteria were identified by the Karmanos Cancer Institute (KCI) Epidemiology Research Core using registry data from the Metropolitan Detroit Cancer Surveillance System (MDCSS). ER and PR status was recorded in MDCSS data while HER2 status was determined by pathology review for cases diagnosed prior to 2010 and using MDCSS data for cases diagnosed 2010 or later. Tumor blocks were identified and retrieved by the Karmanos Cancer Institute Biobanking and Correlative Sciences Core. Clinical data (stage, grade, age at diagnosis), treatment data (surgery type, first line systemic therapy type, radiation, sequence of surgery and first line systemic therapy), and outcomes data (vital status at last contact, cause of death, and active follow-up time) were obtained via linkage with the MDCSS registry. Among 239 eligible patients, tumor samples were obtained for 226 of these patients for expression profiling and 155 treatment-naïve tumors were utilized for analysis. This study was approved for exemption by Wayne State University Institutional Review Board.

### Detroit AA cohort tumor processing & expression profiling

Formalin-fixed paraffin-embedded (FFPE) tumor blocks were processed in two batches. For all tumors, hematoxylin and eosin (H&E) slides were created and unstained tissue curls were cut from four 10mm unstained slides. Tissue curls were generated to correspond to the pathologist-defined tumor area and collected in DNAse/RNAse free microcentrifuge tubes. Batch 1 total RNA (n = 60) was extracted using the QIASymphony Automated system (Qiagen, Germany) and Batch 2 RNA (n = 166) was extracted using the Qiagen RNeasy FFPE Kit according to the manufacturer protocol. All tumors were profiled using Affymetrix Human Gene ST 2.0 arrays after amplification of RNA using the Affymetrix WT Pico Kit (Santa Clara, CA) in two batches (Batch 1 n = 60 tumors, Batch 2 n = 166 tumors). Raw probe intensity data were exported for statistical analysis. Raw and normalized, log-2 transformed expression data and outcomes data for these 226 tumors are publically available in the Gene Expression Omnibus (https://www.ncbi.nlm.nih.gov/geo/) (accession number GSE142102).

### Validation datasets

We downloaded mRNA expression data for 1,084 breast tumors from The Cancer Genome Atlas (TCGA, PanCancer Atlas) and 318 triple negative breast tumors from the Molecular Taxonomy of Breast Cancer International Consortium (METABRIC) using cBioPortal (http://www.cbioportal.org/). We curated relevant datasets from the Gene Expression Omnibus (https://www.ncbi.nlm.nih.gov/geo/) by searching for “breast cancer expression” and “survival” and filtering results to include on entries for human primary breast cancers (non-cell line) with expression profiling by array or high throughput sequencing (n = 14). We then excluded datasets that did not have appropriate survival information (follow-up time or vital status) or included only patients treated with neoadjuvant chemotherapy (n = 9). Finally, we restricted our analyses to include datasets with at least ten triple negative breast cancers, resulting in two usable GEO datasets: GSE35629-GPL1390 and GSE69031. All expression data were downloaded as Z scores from either RNA sequencing data (TCGA: batch normalized/merged from Illumina HiSeq_RNASeqV2 data) or expression microarrays (METABRIC, GEO datasets). Data were not available for *MIR4311* in any of the four validation datasets. For TCGA, corresponding clinical and demographic data were obtained from the Genomic Data Commons Data Portal (https://portal.gdc.cancer.gov/). For METABRIC and GEO datasets, clinical and demographic data were obtained simultaneously with the expression data.

We subset our analyses to 494 TNBC (12 GSE35629-GPL1390, 21 GSE69031, 158 TCGA, 303 METABRIC) defined by ER, PR, and HER2 negative status with available data for survival (vital status, survival time), age, and stage at diagnosis. METABRIC and GEO tumors were considered TNBC when indicated negative by ER, PR, and HER2 status. TCGA Tumors were considered ER negative when negative by IHC staining, PR negative when negative by IHC staining, and HER2 negative when both 1) negative by either IHC or FISH staining and 2) not positive for IHC staining, FISH staining, or copy number status.

### Statistical methods

All statistical analyses were performed in R (https://cran.r-project.org/). Raw probe intensity data from the Detroit AA cohort were normalized separately by batch as implemented by the “rma” function to perform background subtraction, quantile normalization, summarization of probe sets using median-polish, and log_2_-transformation. We evaluated batch effects using principal components analysis as implemented by the “princomp function” (**S1 Fig**). Batch effects were corrected by standardization of probes (subtracting the mean expression value and dividing by standard deviation) by batch (Batch 1 n = 60, Batch 2 n = 166) [25]. There was no evidence for batch effects after standardization (**S1 Fig**). Differences in expression of significant genes in the Detroit AA cohort was evaluated using both t-tests and Wilcox rank sum tests (**S2 Fig**). We selected only the 155 treatment naïve tumors and 27,016 annotated gene transcript probes for analysis, which were combined into a single dataset for subsequent analyses.

For the Detroit AA cohort, overall survival was evaluated in Cox proportional hazards (CoxPH) models as implemented in the “survival” package and all models were adjusted for stage and age at diagnosis. Grade was homogenous in the cohort and surgery type was strongly correlated with stage, so these variables were not included as covariates. For the initial Detroit AA cohort survival analysis of 27,016 genes, nominal p-values were corrected using the false discovery rate (FDR) method using the “p.adjust” function, and FDR-corrected p-values <0.05 were considered statistically significant. We performed additional adjustment for statistically significant genes to evaluate chemotherapy (none vs. adjuvant) and radiation therapy (none vs. adjusted) as potential confounders. We also evaluated breast cancer specific survival for the Detroit AA cohort using CoxPH models adjusting for stage, age at diagnosis, chemotherapy, and radiation therapy.

For the validation cohorts, overall survival was evaluated using CoxPH models adjusting for age and stage. Two TCGA values (Z score>20) for *SPIC* were excluded as outliers, which were from Asian women and exclusion did not substantially affect the overall association. Associations were estimated within each of the four individual validation cohorts using all participants as well as subset to African American and white participants. We then performed meta-analyses of the age- and stage-adjusted effect estimates from the validation cohorts for both *CLCA2* and *SPIC* to obtain a summary effect estimate, 95% confidence intervals, and p-value from tests of heterogeneity of effects for the overall, African American, and white participants. Based on a threshold of p<0.10 for the test of heterogeneity, a fixed effects model was used for all *CLCA2* and *SPIC* meta-analyses except for the *SPIC* overall analysis, where a random effects models was used.

## Results

We evaluated expression of 27,016 well-annotated genes in 155 treatment-naïve TN tumors from the Detroit AA Cohort (**Table 1**). Approximately half of these women were <55 years of age at diagnosis. Almost 90% of tumors were intraductal carcinomas, and the vast majority of women presented with either localized (63.2%) or regional (31.0%) TNBC. Nearly all (96.8%) tumors were either moderately or poorly differentiated. There was considerable variation in treatment decisions, with slightly more women choosing breast-conserving surgery compared to mastectomy. A quarter of women did not receive adjuvant chemotherapy and about a third of women did not undergo adjuvant radiation therapy, although 92% of women received at least one of these treatments. Approximately 36% of the 155 women died during a mean follow-up time of 5.5 years, where ~60% of deaths were due to breast cancer.

**Table 1:** Patient characteristics of 155 treatment-naïve TN tumors in the Detroit AA cohort.

Characteristic	N	%
Age at Diagnosis		
<55 years	81	52.3%
56+ years	74	47.7%
Laterally		
Right	83	53.5%
Left	72	46.5%
Stage		
Localized	98	63.2%
Regional	48	31.0%
Distant	9	5.8%
Histology		
Intraductal carcinoma	136	87.7%
Adenocarcinoma	7	4.5%
Lobular carcinoma	1	0.6%
Other^a^	11	7.1%
Grade		
I-Well differentiated	1	0.6%
II-Moderately differentiated	21	13.5%
III-Poorly differentiated	129	83.2%
IV-Undifferentiated	2	1.3%
Unknown	2	1.3%
Surgical therapy		
Breast-conserving	88	56.8%
Mastectomy	64	41.3%
Unknown	3	1.9%
Adjuvant chemotherapy		
No	40	25.8%
Yes	115	74.2
Adjuvant radiation therapy		
No	55	35.5%
Yes	100	64.5%
Status		
Alive	99	63.9%
Dead (breast cancer)	33	21.3%
Dead (other cause)	23	14.8%
	**Mean**	**Std.**
Age at Diagnosis (years)	55.4	13.4
Follow-up time (months)	65.9	40.3

^a^Includes 2 adenoid cystic carcinomas, 1 atypical medullary carcinoma, 2 invasive micropapillary carcinomas, 4 medullary carcinomas, and 2 metaplastic carcinomas

**Fig 1:**
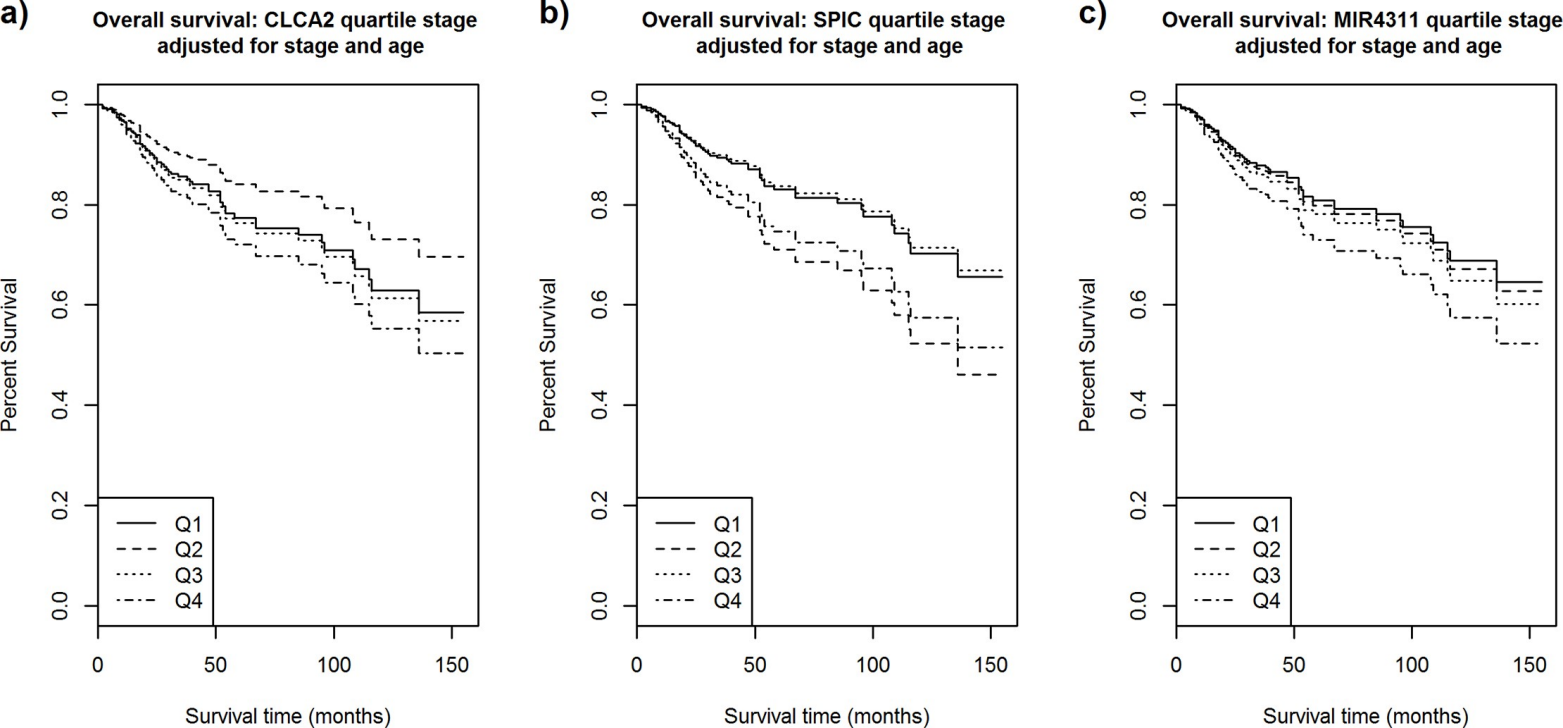
Kaplan-Meier survival curves by CLCA2, SPIC, and MIR4311 quartile in the Detroit AA cohort. Kaplan−Meier plots for overall survival by (a) CLCA2 quartile, (b) SPIC quartile, and (c) MIR4311 quartile among 155 treatment-naïve TN tumors (56 deaths) in the Detroit AA cohort. Solid lines represent curves for Q1, dashed lines represent curves for Q2, dotted lines represent curves for Q3, and dash-dotted lines represent curves for Q4 in each panel.

Among the 27,016 genes evaluated for association with overall survival adjusting for stage and grade (**S1 Table**), three genes remained statistically after FDR correction (FDR p<0.05) (**Table 2, Fig 1**). *CLCA2* (Chloride Channel Accessory 2) expression was associated with a 56% increased risk of death [Hazard ratio (HR) = 1.56, 95% confidence interval (CI) 1.31–1.86, nominal p = 5.1x10^-7^, FDR p = 0.014], *SPIC* (Spi-C Transcription Factor) expression was associated with a 47% increase in mortality [95%CI 1.26–1.73, nominal p = 1.8x10^-6^, FDR p = 0.022], and *MIR4311* expression was associated with a 59% increased risk of death [95% CI 1.31–1.92, nominal p = 2.5x10^-5^, FDR p = 0.022]. Additional adjustment for adjuvant chemotherapy and radiation did not change the effect estimates for any of these three genes (**Table 2**). We also evaluated associations with breast cancer-specific survival using the fully adjusted model for stage, age, and treatment (**Table 2**). While statistical significance was slightly reduced due to the decrease in the number of events (33 breast cancer deaths compared to 55 deaths from any cause), the effects estimates for all three genes remained stable compared to the overall survival analysis [*CLCA2* HR = 1.66, *SPIC* HR = 1.52, *MIR4311* HR = 1.52].

**Table 2:** Associations between *CLCA2*, *SPIC*, and *MIR4311* and overall and breast cancer specific survival in the Detroit AA cohort.

	Overall survival- Stage & Age adjusted				Overall survival- Add chemo & radiation				Breast-cancer specific survival- Fully adjusted			
Characteristics	HR	LCL	UCL	P—value	HR	LCL	UCL	P—value	HR	LCL	UCL	P—value
CLCA2 (16666755)	1.56	1.31	1.86	5.1x10^-7^	1.64	1.36	1.98	3.1x10^-7^	1.66	1.30	2.14	5.8x10^-5^
Stage												
Local	1.00	(ref)			1.00	(ref)			1.00	(ref)		
Regional	1.60	0.89	2.85	0.11	1.81	0.99	3.28	0.051	2.52	1.18	5.42	0.018
Distant	22.17	8.51	57.80	2.3X10^-10^	20.92	7.85	55.74	1.19x10^-9^	13.1	3.68	46.60	7.0x10^-5^
Age	1.03	1.01	1.06	0.0016	1.02	0.99	1.04	0.0724	0.99	0.96	1.02	0.55
Chemotherapy												
None					1.00	(ref)			1.00	(ref)		
Adjuvant					0.44	0.23	0.84	0.012	0.44	0.78	1.07	0.071
Radiation												
None					1.00	(ref)			1.00	(ref)		
Adjuvant					0.72	0.42	1.25	0.24	0.97	0.46	2.05	0.95
SPIC (16755826)	1.47	1.26	1.73	1.8x10^-6^	1.47	1.25	1.73	2.9x10^-6^	1.52	1.21	1.92	3.9x10^-4^
Stage												
Local	1.00	(ref)			1.00	(ref)			1.00	(ref)		
Regional	1.64	0.91	2.98	0.10	1.79	0.98	3.26	0.056	2.09	0.96	4.56	0.064
Distant	24.85	8.26	57.80	5.2x10^-10^	19.63	7.36	52.35	2.7x10^-9^	10.28	2.92	36.17	2.8x10^-4^
Age	1.04	1.02	1.07	1.4x10^-4^	1.03	1.01	1.06	6.8x10^-3^	1.00	0.97	1.03	0.82
Chemotherapy												
None					1.0	(ref)			1.00	(ref)		
Adjuvant					0.53	0.28	0.99	0.045	0.58	0.23	1.42	0.23
Radiation												
None					1.00	(ref)			1.00	(ref)		
Adjuvant					0.64	0.37	1.11	0.11	0.82	0.39	1.73	0.61
MIR4311 (16802160)	1.59	1.31	1.92	2.5x10^-6^	1.6	1.31	1.95	3.3x10^-6^	1.52	1.21	1.92	4.0x10^-4^
Stage												
Local	1.00	(ref)			1.00	(ref)			1.00	(ref)		
Regional	1.51	0.83	2.75	0.18	1.66	0.91	3.05	0.099	2.09	0.96	4.56	0.064
Distant	20	7.84	53.49	2.4x10^-9^	17.81	6.66	47.62	9.6x10^-9^	10.28	2.92	36.17	2.8x10^-4^
Age	1.04	1.02	1.06	1.9x10^-4^	1.03	1.01	1.06	5.3x10^-3^	1.00	0.97	1.03	0.82
Chemotherapy												
None					1.00	(ref)			1.00	(ref)		
Adjuvant					0.6	0.31	1.14	0.12	0.58	0.23	1.42	0.23
Radiation												
None					1.00	(ref)			1.00	(ref)		
Adjuvant					0.57	0.33	0.99	0.046	0.82	0.39	1.73	0.61

**Fig 2:**
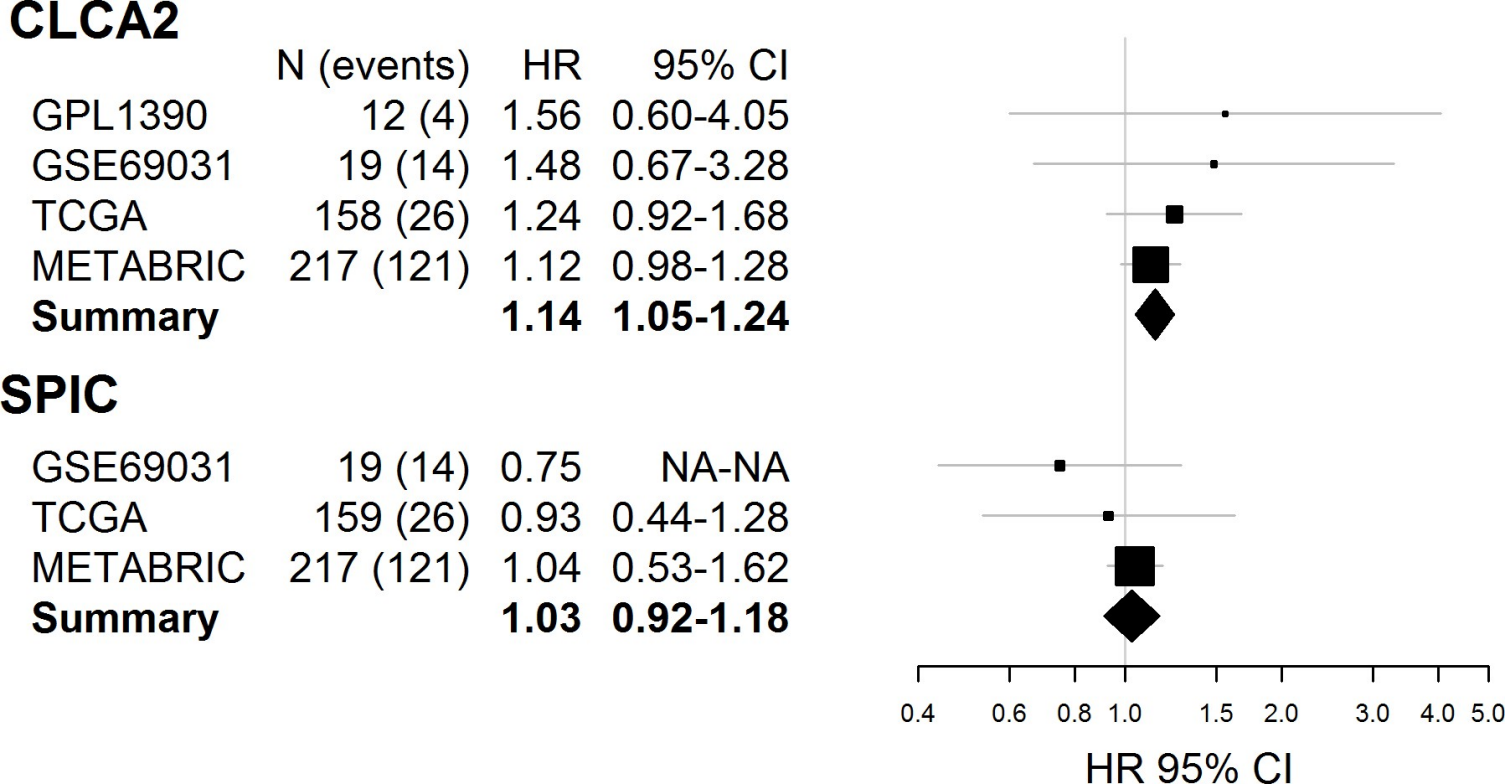
Forest plots for validation cohort analyses of *CLCA2*, *SPIC*, and overall survival. Forest plots for analyses of GSE35629-GPL1390, GSE69031, TCGA, and METABRIC are shown for *CLCA2* and *SPIC*. Only overall, rather than race-specific, analyses are shown because of small sample size (GPL1390, GSE69031) or lack of race data (METABRIC). All estimates are adjusted for age and stage at diagnosis. Study-specific hazard ratios (HR) are denoted by black boxes and 95% confidence intervals (CI) are denoted by corresponding black lines. Box heights are inversely proportional to precision of the HR estimate as influenced by sample size, such that a larger HR box indicates larger sample size and better precision. Summary estimates are denoted as diamonds, where the width of the diamond corresponds to the 95% CI. Estimates with confidence intervals that do not overlap the null line at 1.0 indicate significance at p<0.05.

We next sought to validate these associations using publically available expression data from TN breast tumors in TCGA (n = 158), METABRIC (n = 303), GSE35629-GPL1390 (n = 12), and GSE69031 (n = 21). Only *CLCA2* expression data was available for all four validation cohorts, and *SPIC* expression data was available for only three (GSE69031, TCGA, METABRIC). While *CLCA2* was not significantly associated with survival in the four individual validation datasets, the magnitude and direction of effect were consistent with the association observed for *CLCA2* in the Detroit cohort (**Fig 2**). Indeed, *CLCA2* was significantly associated with overall survival in the validation meta-analysis [HR = 1.14, 95% CI 1.05–1.24, p = 0.038, p-heterogeneity = 0.88]. *SPIC* was not significantly associated with overall survival in individual analyses or in the meta-analysis. We were only able to estimate race-specific hazard ratios using TCGA data, which has a substantially reduced AA sample size and number of deaths compared to the Detroit AA cohort (54 AA cases, 11 deaths). While not statistically significant, the hazard ratio estimates for both *CLCA2* and *SPIC* were comparable to those seen in the Detroit AA cohort [*CLCA2*: HR = 1.41, 95% CI 0.77–2.58; *SPIC*: HR = 1.24, 95% CI 0.52–2.97]. The *CLCA2* association with survival was similar among European Americans in TCGA (92 EA cases, 13 deaths) [HR = 1.26, 95% CI 0.82–1.93], while *SPIC* was associated with a non-significant reduction in death among European Americans [HR = 0.46, 95% CI 0.063–3.31].

## Discussion

Here we evaluated potential prognostic biomarkers for TNBC by analyzing associations between transcriptome-wide tumor expression profiles and survival in 155 treatment-naïve tumors from AA women with TNBC in Detroit. Three genes (*CLCA2*, *SPIC*, *MIR4311*) were associated with overall and breast cancer specific survival in this discovery cohort. While associations between *CLCA2* and *SPIC* in validation cohorts were not statistically significant, meta-analysis revealed that *CLCA2* was significantly associated with overall survival when combining four TNBC validation cohorts.

*CLCA2* encodes a calcium-activated chloride channel regulator family member, proteins that regulate the transport of chloride across the plasma membrane. *CLCA2* confers an antiproliferative role, where expression is upregulated by the tumor suppressor protein p53 in response to DNA damage [26]. *CLCA2* expression has been found to be downregulated in breast tumors, whereas *CLCA2* expression is known to inhibit migration and invasion while simultaneously promoting mesenchymal-to-epithelial transition in cancer cell lines [27–30]. Interestingly, we found that increased expression of this putative tumor suppressor was associated with worse survival. Despite this *in vitro* evidence for a favorable prognostic role of *CLCA2* in cancers, very few studies have been reported associations between *CLCA2* expression in cancer with respect to clinical outcomes in humans [31, 32]. The one study that reported associations between *CLCA2* and survival in cancer reported worse disease-free survival associated with increased *CLCA2* expression in early stage lung adenocarcinoma [31], which is comparable to our findings. While the *CLCA2* has clear implications for invasion and metastasis in breast cancer, the mechanism by which increased *CLCA2* tumor expression may be related to worse clinical outcomes for women with TNBC is unclear.

*SPIC* is a transcription factor that controls the development of red pulp macrophages, splenic macrophages which are critical for blood homeostasis via red blood cell recycling and iron homeostasis [33]. *SPIC* has also been shown to act as a lymphoid-specific enhancer and regulates *VCAM1* [34], a gene that has been associated with progression, angiogenesis, and metastasis in breast cancer [35]. *VCAM1* is also critical for macrophage-mediated retention of hematopoietic stem cells in the spleen, and there is evidence that these splenic stem cells are a continuous source of tumor associated macrophages throughout tumor progression [36]. *SPIC* is also involved in the genomic stability of pre-B cells, where DNA double strand breaks were found to inhibit pre-B cell receptor signaling through induction of *SPIC* [37]. While few reports of the relevance of *SPIC* to cancer development or progression exist, one study found that *SPIC* transcription factor binding sites were enriched among lncRNAs found to be involved in the neuroendocrine transdifferentiation process through which Treatment-induced neuroendocrine prostate cancers arise [38]. Given the known importance of tumor infiltrating lymphocytes to breast cancer overall and TNBC specifically [39–41], the mechanisms relating *SPIC*, tumor associated macrophages, and B cells to tumor progression should be further explored.

Much less is known about the function or expression targets of *MIR4311*, a microRNA gene located on chromosome 15q22. However, using the NCBI Phenotype-Genotype Integrator (https://www.ncbi.nlm.nih.gov/gap/phegeni), an intergenic variant in the MIR4311/DIS3L region (rs6494560) was associated with obesity (4.917 x 10–6), waist circumference (4.798 x 10–5), and body mass index (4.863 x 10–5) in the Family Heart Study genome wide association study [42]. Obesity is a known risk factor for TNBC [43], and there is some evidence that overweight or obesity is related to TNBC progression [44].

Here we identified *CLCA2* as a potential prognostic marker for TNBC. While we were somewhat limited in our ability to validate race-specific findings in TCGA due to small race-specific sample sizes and lack of treatment data, we were able to show that *CLCA2* is associated with increased risk of death for TNBC considering the discovery and validation data sets. We were also unable to directly evaluate racial comparisons in effects in our Detroit cohort due to our selection criteria, which will be important to evaluate in future studies. It is also possible due to limited sample size that we were unable to detect associations between expression of additional important genes and survival. Nevertheless, our findings suggest potential new mechanisms for TN tumor progression and identify possible new therapeutic targets that are relevant for AA women.

## Supporting information

S1 Table Associations between 27,016 annotated genes and survival in the Detroit AA cohort. (XLSX)

S2 Table Associations between CLCA2 and SPIC expression and stage in TCGA. (XLSX)

S1 Fig Principal components analysis of batch effects in Detroit AA cohort. Plots of the first two principal components are shown pre- and post-standardization. Red dots correspond to batch 1 samples and black dots correspond to batch 2 samples.
(TIFF)

S2 Fig Boxplots of *CLCA2*, *SPIC*, and *MIR4311* expression by batch. Boxplots of CLCA2, SPIC, and MIR4311 expression are shown by batch. The bold line within each boxplot represents median expression, and the upper and lower bounds of the boxplot represent the 75th and 25th percentiles, respectively. Outer edges reflect the 10th (lower) and 90th (upper) percentiles, respectively, and outliers are denoted in shaded circles.
(TIFF)
