# Supplementary figures and images for: CLCA2 expression is associated with survival among African American women with triple negative breast cancer
Source: PLoS One. 2020 Apr 16;15(4):e0231712. doi: 10.1371/journal.pone.0231712 (PMC7161959; doi:10.1371/journal.pone.0231712)

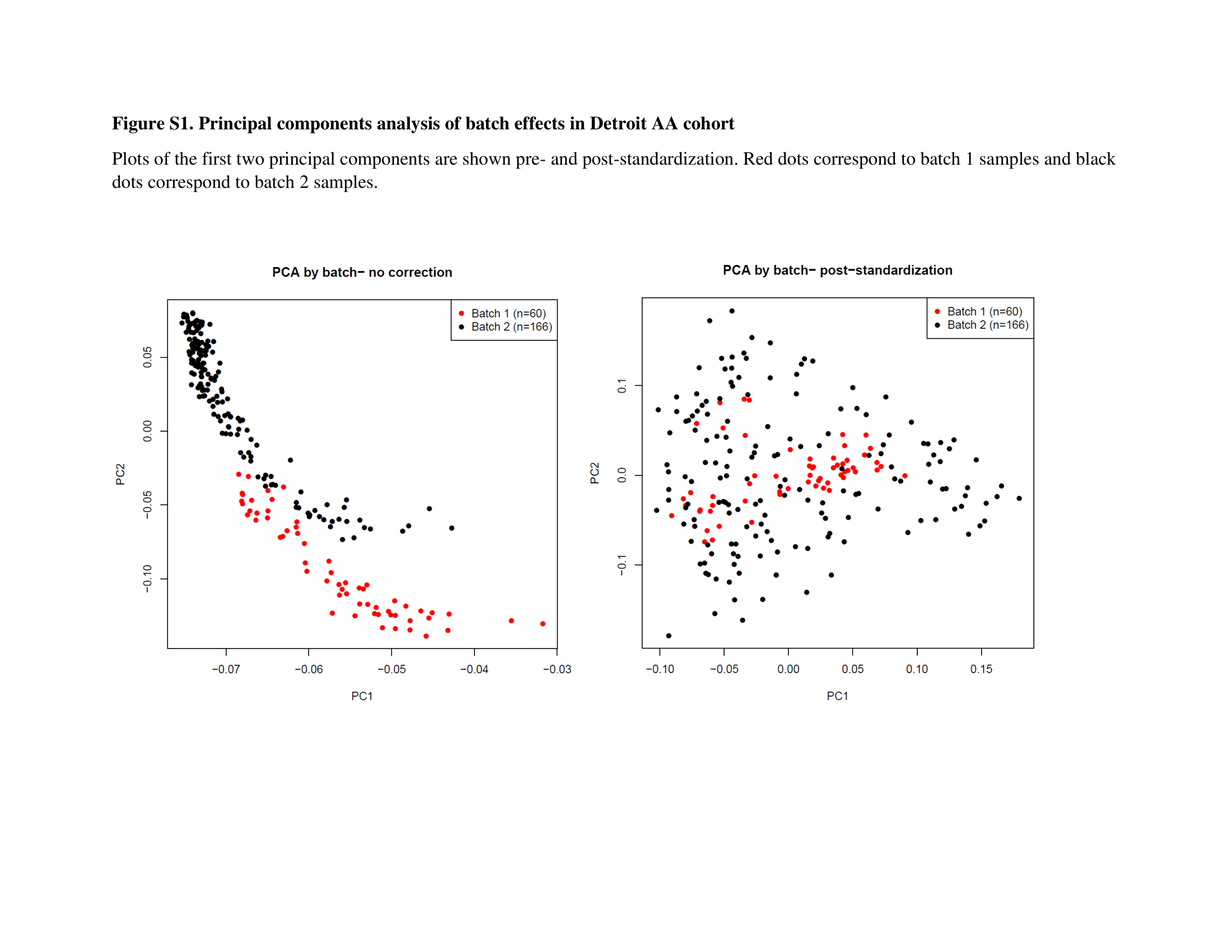

Supplement: S1 Fig — Plots of the first two principal components are shown pre- and post-standardization. Red dots correspond to batch 1 samples and black dots correspond to batch 2 samples. (TIFF) [file pone.0231712.s003.tiff]

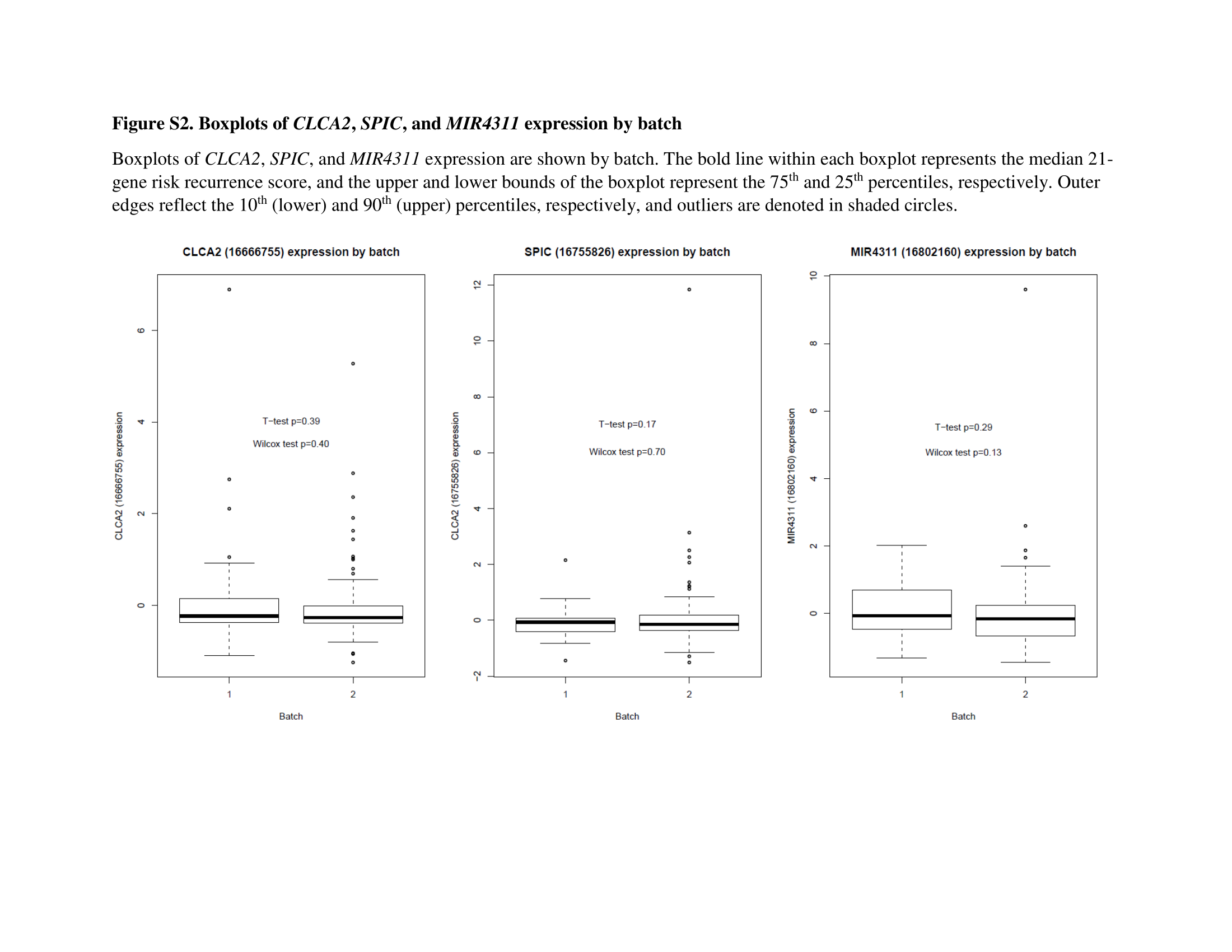

Supplement: S2 Fig — Boxplots of CLCA2, SPIC, and MIR4311 expression are shown by batch. The bold line within each boxplot represents median expression, and the upper and lower bounds of the boxplot represent the 75th and 25th percentiles, respectively. Outer edges reflect the 10th (lower) and 90th (upper) percentiles, respectively, and outliers are denoted in shaded circles. (TIFF) [file pone.0231712.s004.tiff]
